# Trends in the incidence, survival, and prognostic nomogram of angiosarcoma in the United States

**DOI:** 10.1097/MD.0000000000041152

**Published:** 2025-01-03

**Authors:** Dong Zeng, Zhiyi Wang, Yongdong Feng, Michael J. McKay, Monika K. Masanam, Haixia Long, Xi Cao

**Affiliations:** a Institute of Cancer, Xinqiao Hospital, Third Military Medical University, Chongqing, China; b Northern Cancer Service, Northwest Regional Hospital, Burnie, Tasmania, Australia; c Rural Clinical School, University of Tasmania, Burnie, Tasmania, Australia; d Department of Surgery, MedStar Georgetown University Hospital, Washington, DC; e Department of Medical Engineering, Xinqiao Hospital, Third Military Medical University, Chongqing, China.

**Keywords:** angiosarcoma, incidence, nomogram, prognosis

## Abstract

This study aimed to investigate the epidemiological trends of angiosarcoma and to establish a tool to estimate its prognosis. Data from the Surveillance, Epidemiology, and End Results (SEER) database (1975–2016) were used to assess trends in the epidemiology of angiosarcoma, and a nomogram was established based on independent prognostic factors. The age-adjusted incidence of angiosarcoma gradually increased from 0.13/100,000 in 1975 to 0.33/100,000 in 2016 (annual percentage change [2.4]). The most significant increase was observed in patients aged ≥ 60 years. The same increasing trend was observed across all the stages and grades. The limited-duration prevalence increased from 0.0003% in 1992 to 0.0013% in 2016 (*P* < .05). In multivariable analyses, age, sex, marital status, grade, historical stage, surgery, site, and tumor size were independent prognostic factors for angiosarcoma. The concordance index of the nomogram was significantly higher than that of the American Joint Committee on Cancer (AJCC) 6th edition and the AJCC 7th edition (0.74 vs 0.61 vs 0.66, respectively). Calibration analysis showed optimal agreement between nomogram predictions and actual observations. The incidence and prevalence of angiosarcoma has increased over the past 40 years. We established a nomogram to predict the overall survival of patients with angiosarcoma.

## 1. Introduction

Angiosarcoma is a rare and highly aggressive malignant tumor. It accounts for <2% of all soft tissue sarcomas.^[1–3]^ Angiosarcoma can originate from many sites in the body, such as soft tissue, skin, breast, heart, liver, bone, and spleen^[4–8]^; therefore, the heterogeneity among angiosarcomas is considerable. Most angiosarcomas spontaneously develop. However, there are several well-established risk factors in the literature, including radiation therapy, chronic lymphedema, familial syndromes, and exposure to chemical agents such as vinyl chloride, thorium dioxide, arsenic and radium.^[9,10]^ Angiosarcomas have poor prognosis. The median survival time is approximately 30 to 50 months, with a 5-year survival rate of 10% to 50%.^[11–13]^ Owing to the rarity of these tumors, most previous studies have been limited to small institutional series, and many have been case reports without epidemiological or multivariable analyses. Therefore, the current epidemiological data are lacking. However, the incidence and prevalence of angiosarcomas remain unclear. In addition, the American Joint Committee on Cancer (AJCC) tumor-node-metastasis staging system is used to predict prognosis^[14]^; however, accurate prognostic models of angiosarcoma have not yet been established because of the rarity of the disease and heterogeneity among studies. In this study, we comprehensively analyzed epidemiological trends, clinical characteristics, prognostic factors, and prognostic models in the United States from 1975 to 2016 using the Surveillance, Epidemiology, and End Results (SEER) database. The findings of this study will benefit clinical practice and future research in this area.

## 2. Materials and methods

### 2.1. Ethic statement

All the procedures performed in this study were conducted in accordance with the Declaration of Helsinki. All patient information was publicly available; therefore, institutional review board approval was not required.

### 2.2. Data source

The SEER database is a publicly available cancer reporting system updated annually. It was initiated in 1973, is population-based, and contains information from 18 states.^[15]^ We retrieved data from the SEER 18 database, which was released in August 2019, using the SEER*Stat software (version 8.3.6; https://seer.cancer.gov/seerstat/). The SEER 9, 13, and 18 databases, cover approximately 9.4%, 13.4%, and 27.8% of the incident cases in the United States, respectively. To maximize the representation of our research, we calculated the 1973 to 1991 incidences with SEER 9, the 1992 to 1999 incidences with SEER 13, and the 2000 to 2016 incidences with SEER 18. As patient information was de-identified and publicly available, institutional review board approval was not required.

Patients were diagnosed with angiosarcoma as defined by the International Classification of Diseases for Oncology, 3rd edition (ICD-O-3), with the histological code hemangiosarcoma 9120. For the survival analysis, only patients with specific prognostic data were included.

### 2.3. Angiosarcoma classification

Demographic and clinical information, such as age, sex, year of diagnosis, race, marital status, historic stage, grade, site recode (ICD-O-3 site), clinical tumor size, surgery, AJCC stage 7th edition, AJCC stage 6th edition, and vital status records, were extracted. Age was categorized as <30 years, 30 to 59 years, and ≥60 years. Year of diagnosis was classified as 1975 to 1987, 1988 to 2000, 2001 to 2005, 2006 to 2010, and 2011 to 2016. Race was categorized as White, Black, or others, with others comprising American Indian/AK Native, Asian/Pacific Islander, and others. Marital status was coded as married, unmarried, or unknown. The unmarried group included single, separated, divorced, and widowed individuals. We used historic stage information to classify cases as local, regional, distant, or unknown. Angiosarcoma of the breast is traditionally graded using a 3-tier system and classified as low, intermediate, or high-grade based on cytologic atypia, mitotic count, extent of solid areas, and necrosis.^[16]^ For the purpose of this analysis, the tumor grade was coded as grade (G)1, well differentiated; G2, moderately differentiated; G3, poorly differentiated; G4, undifferentiated; and unknown, according to the AJCC guidelines. Six sites were selected, based on the number of cases. These sites were soft tissues including the heart (C380, C470-479, C490-C499); skin excluding basal and squamous (C440-C449); breast (C500-C509); liver (C220); bones and joints (C400-C419); and lungs, bronchi, trachea, mediastinum, and other respiratory organs (C340-C349, C339, C381-C383, C388, C390, C398, and C399, respectively). Clinical tumor sizes were classified as <5 cm, ≥5 cm, or unknown. The surgical information was coded as yes, no, or unknown. The AJCC stage groups were defined as 1, 2, 3, or 4.

### 2.4. Statistical analysis

All incidence rates and limited-duration prevalence rates were age-adjusted to the 2000 US standard population; they were expressed per 100,000 person-years and calculated using the SEER*Stat software, version 8.3.6 (Surveillance Research Program, National Cancer Institute). The annual percentage change (APC) was calculated using log-linear regression, and the change in incidence was assessed using the Joinpoint Regression Program version 4.7.0 (Surveillance Research Program, National Cancer Institute).^[17]^ Graphs were created using GraphPad Prism, version 7.02 (GraphPad Software, San Diego).

Univariate and multivariate survival analyses of the SEER 18 data were performed to assess the most recent trends in survival. SPSS software (version 20.0; IBM Corp., Armonk) was used for the survival analysis. Kaplan–Meier curves were constructed to estimate overall survival (OS), and log-rank tests were used to determine the significance of differences. Cox regression analysis was performed for multivariate analysis.

A nomogram was formulated based on the results of multivariate analysis using R software (version 3.6.1; https://www.r-project.org). The performance of the nomogram includes discrimination and calibration. Discrimination was assessed using the concordance index (C-index). Calibration curves were generated to compare the predicted survival rates with the actual survival rates, using the Kaplan–Meier method. Comparisons between the nomogram and AJCC staging system were made using the R software and evaluated using the C-index. A higher C-index indicates more accurate prognostic prediction.^[18]^ Statistical significance was set at *P* < .05.

## 3. Results

### 3.1. Baseline characteristics

As shown in Table 1, 5033 patients were included in this study. Of these patients, 2702 (53.7%) were female, and 69.1% were older than 60 years. Of the 4044 patients with a known stage, 1915 had local disease, 1152 had regional disease, and 937 had distant metastasis. Regarding the grade of 2467 patients with known data, 260 had G1, 399 had G2, 857 had G3, and 951 had G4. The 3 sites with the highest incidence were soft tissue including the heart, skin excluding basal and squamous cells, and breast, accounting for 43.3%, 17.8%, and 13.3%, respectively.

**Table 1:** Baseline characteristics of patient diagnosis with angiosarcoma from 1975 to 2016.

Factors	Group	N (%)
Age (yr)	<30	227 (4.51)
	30–59	1330 (26.43)
	≥60	3476 (69.06)
Gender	Male	2331 (46.31)
	Female	2702 (53.69)
Marital status	Married	2677 (53.19)
	Unmarried	2021 (40.15)
	Unknown	335 (6.66)
Race	Black	4213 (83.71)
	White	392 (7.79)
	Other	428 (8.5)
Historic stage	Local	1915 (38.05)
	Regional	1152 (22.89)
	Distant	937 (18.62)
	Unknown	1029 (20.45)
Grade	Well	260 (5.17)
	Moderately	399 (7.93)
	Poorly	857 (17.03)
	Undifferentiated	951 (18.9)
	Unknown	2566 (50.98)
Site	Soft tissue including heart	2179 (43.29)
	Skin excluding basal and squamous	897 (17.82)
	Breast	671 (13.33)
	Liver	366 (7.27)
	Bones and joints	152 (3.02)
	Lung, bronchus	139 (2.76)
	Other	629 (12.5)
Tumor size	<5 cm	934 (18.56)
	≥5 cm	926 (18.4)
	Unknown	3173 (63.04)
Surgery	Yes	3275 (65.07)
	No	1704 (33.86)
	Unknown	54 (1.07)

### 3.2. Incidence trend and prevalence

**Figure 1.**
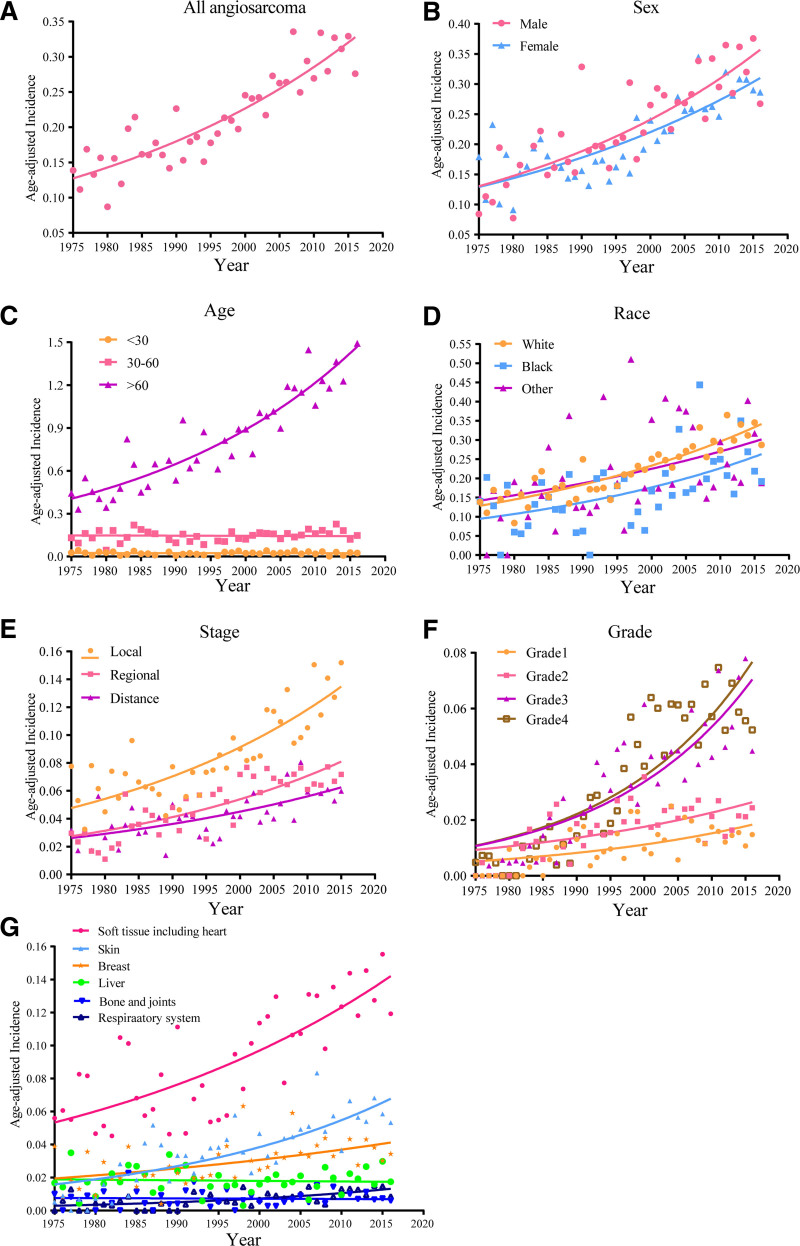
Annual age-adjusted incidence of angiosarcoma. (A) Total age-adjusted incidence of angiosarcoma. (B) By sex. (C) By age. (D) By race. (E) By stage. (F) By grade. (G) By sites.

From 1975 to 2016, the age-adjusted incidence of angiosarcoma increased significantly from 0.13/100,000 to 0.33/100,000 (APC, 2.4; 95% confidence interval [CI]: 1.9–2.8; *P* < .0; Fig. 1A). Both males and females showed similar increasing trends (Fig. 1B). We calculated the age-adjusted incidence in 3 age groups: 30, 30 to 5, and 60 years. As shown in Figure 1C, the most significant increase was observed in patients aged 60 years (APC, 3.4; 95% CI: 2.9–3.8; *P* < .05). In those aged 30 to 59 years, the incidence changed from 0.12 per 100,000 to 0.16 per 100,000 (APC, 0.8; 95% CI: 0–1.5; *P* < .05), whereas no obvious change was observed in patients younger than 30 years (APC, 0.1; *P* > .05). Among the racial groups, the incidence increased the most in White individuals, from 0.13 per 100,000 persons to 0.34 per 100,000 persons (Fig. 1D; APC, 2.5; 95% CI: 2.0–2.9; *P* < .05). The same increasing trend was observed across all stages and grades (Fig. 1E, F). The rate of local angiosarcoma increased from 0.05 per 100,000 to 0.13 per 100,000 individuals (*P* < .05). Among the grades, the incidence increased the most for G4 angiosarcoma, from 0.01 per 100,000 persons to 0.13 per 100,000 persons (APC, 7.3; 95% CI: 6.0–8.0; *P* < .05). The increase in the incidence occurred in some sites, especially in soft tissue including the heart (APC, 2.3; 95% CI: 1.7–3.0; *P* < .05), skin excluding basal, squamous, and breast skin areas (APC, 5.1; 95% CI: 3.8–6.5; *P* < .05), and breast (APC, 2.4; 95% CI: 1.2–3.6; *P* < .05; Fig. 1G).

**Figure 2.**
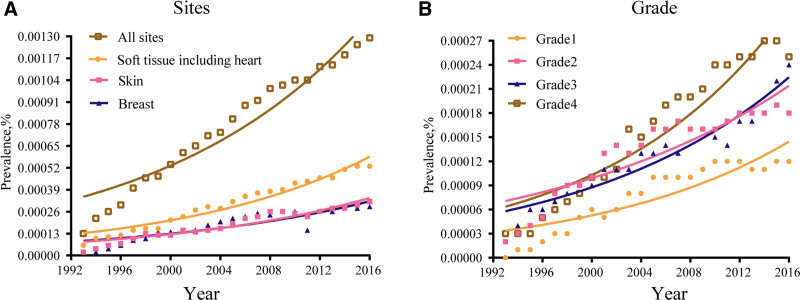
The limited-duration prevalence of angiosarcoma. (A) By sites. (B) By grade.

As shown in Figure 2A, the limited-duration prevalence increased from 0.0003% in 1992 to 0.0013% in 2016 (*P* < .05). Among the sites, soft tissues, including the heart, had the highest prevalence. Among the grades, the most obvious increase was observed in grade 4 (Fig. 2B).

### 3.3. Survival

The median OS was 16 months and the 1-, 3-, and 5-year survival rates were 55.2%, 33.7%, and 26.4%, respectively. When we analyzed the prognostic factors in the univariate analysis, sex, age, marital status, year of diagnosis, historic stage, grade, tumor size, surgery, and site were prognostic factors (Fig. S1, Supplemental Digital Content, http://links.lww.com/MD/O253).

**Figure 3.**
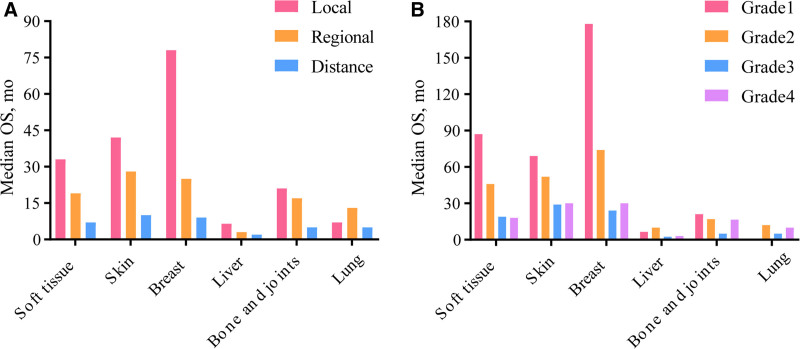
The median OS of angiosarcoma. (A) By sites and stage. (B) By sites and grade. OS = overall survival.

Next, we analyzed survival patterns according to site and stage. As shown in Figure 3A, the median OS ranged from 6.5 to 78 months in patients with local disease. Patients with angiosarcoma of the breast had the best prognosis, whereas those with angiosarcoma of the liver had the worst. Among the patients with regional disease and distant metastasis, those with angiosarcoma of the skin, excluding basal and squamous cells, had the best median OS, whereas those with angiosarcoma of the liver had the worst median OS. We then evaluated OS according to site and grade (Fig. 3B). Patients with angiosarcoma of the breast had the longest median OS among those with stages G1 and G2. In general, patients with G3 and G4 angiosarcomas had a poor OS, ranging from 2.5 to 30 months.

We then carried out multivariable analysis. Age, sex, marital status, grade, historical stage, surgery, site, and tumor size were independent prognostic factors for angiosarcoma (Table 2). Compared to patients aged <30 years, those aged ≥60 years had a worse OS (hazard ratio [HR], 2.594; 95% CI: 2.185–3.080; *P* < .05). Unmarried patients had worse OS than married patients (HR, 1.226; 95% CI: 1.143–1.314; *P* < .05). In general, patients with local and stage G1 disease have better survival rates. Overall, patients benefited from surgery (HR, 1.73; 95% CI: 1.597–1.874; *P* < .05). In addition, patients with angiosarcoma of the liver had the worst OS, and tumor size >5 cm was a negative prognostic factor.

**Table 2:** Multivariate analyses of overall survival in angiosarcoma.

Factors	Group	Hazard ratio (95% CI)	*P* value
Sex	Male/female	0.828 (0.772–0.888)	0
Age (yr)	<30	–	0
	30–59	1.430 (1.198–1.707)	0
	≥60	2.594 (2.185–3.080)	0
Year of diagnosis	1975–1987	–	.422
	1988–2000	1.024 (0.890–1.178)	.743
	2001–2005	1.065 (0.925–1.226)	.382
	2006–2010	1.103 (0.953–1.275)	.189
	2011–2016	1.023 (0.883–1.184)	.765
Race	White	–	.27
	Black	1.051 (0.933–1.185)	.409
	Other	0.923 (0.745–0.994)	.19
Marital status	Married	–	0
	Unmarried	1.226 (1.143–1.314)	0
	Unknown	0.861 (0.745–0.994)	.041
Historic stage	Local	–	0
	Regional	1.394 (1.279–1.520)	0
	Distance	2.501 (2.269–2.756)	0
	Unknown	1.388 (1.250–1.541)	0
Grade	Well	–	0
	Moderately	1.191 (0.959–1.180)	.114
	Poorly	1.939 (1.599–2.351)	0
	Undifferentiated	1.936 (1.599–2.343)	0
	Unknown	1.782 (1.484–2.141)	0
Tumor size	<5 cm	–	0
	≥5 cm	1.355 (1.210–1.517)	0
	Unknown	1.316 (1.184–1.462)	0
Surgery	Yes	–	0
	No	1.730 (1.597–1.874)	0
	Unknown	1.593 (1.178–2.154)	.002
Site	Soft tissue including heart	–	0
	Skin excluding basal and squamous	0.779 (0.708–0.857)	0
	Breast	0.821 (0.731–0.923)	.001
	Liver	2.741 (2.417–3.108)	0
	Bone and joints	1.218 (1.009–1.471)	.04
	Lung and bronchus	1.420 (1.178–1.711)	0
	Other	1.549 (1.396–1.719)	0

### 3.4. Prediction model

**Figure 4.**
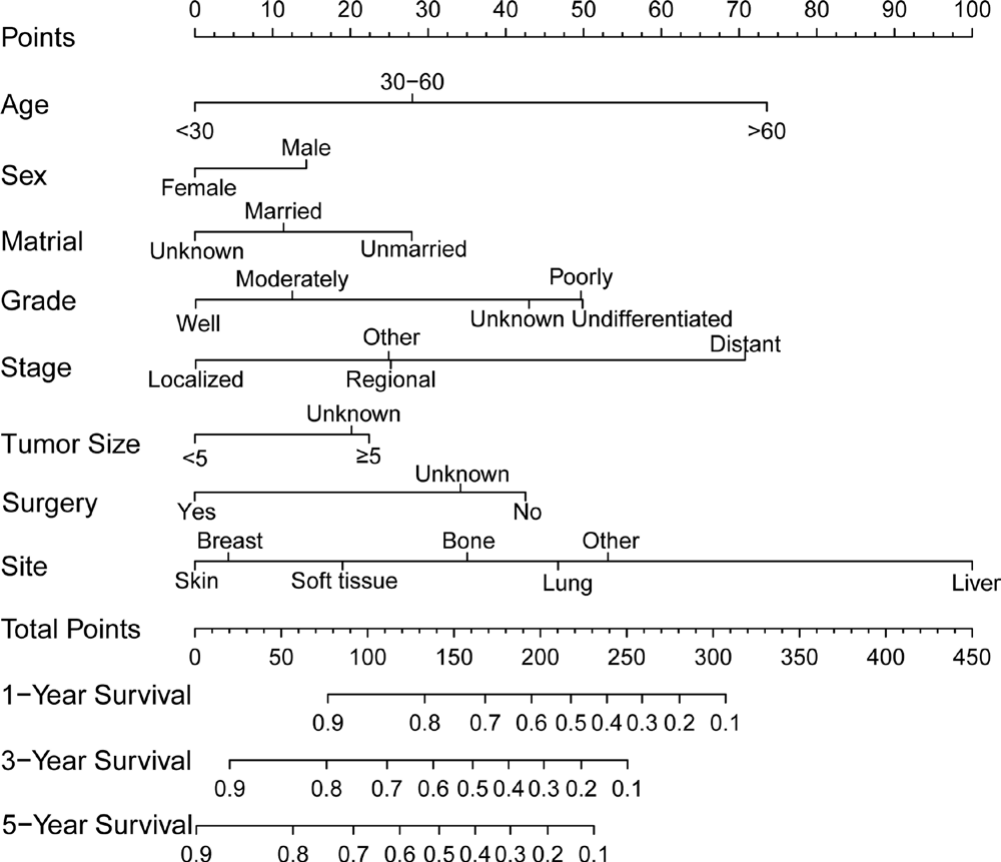
Nomograms to predict the 1-, 3-, and 5-year OS of patients with angiosarcoma. OS = overall survival.

**Figure 5.**
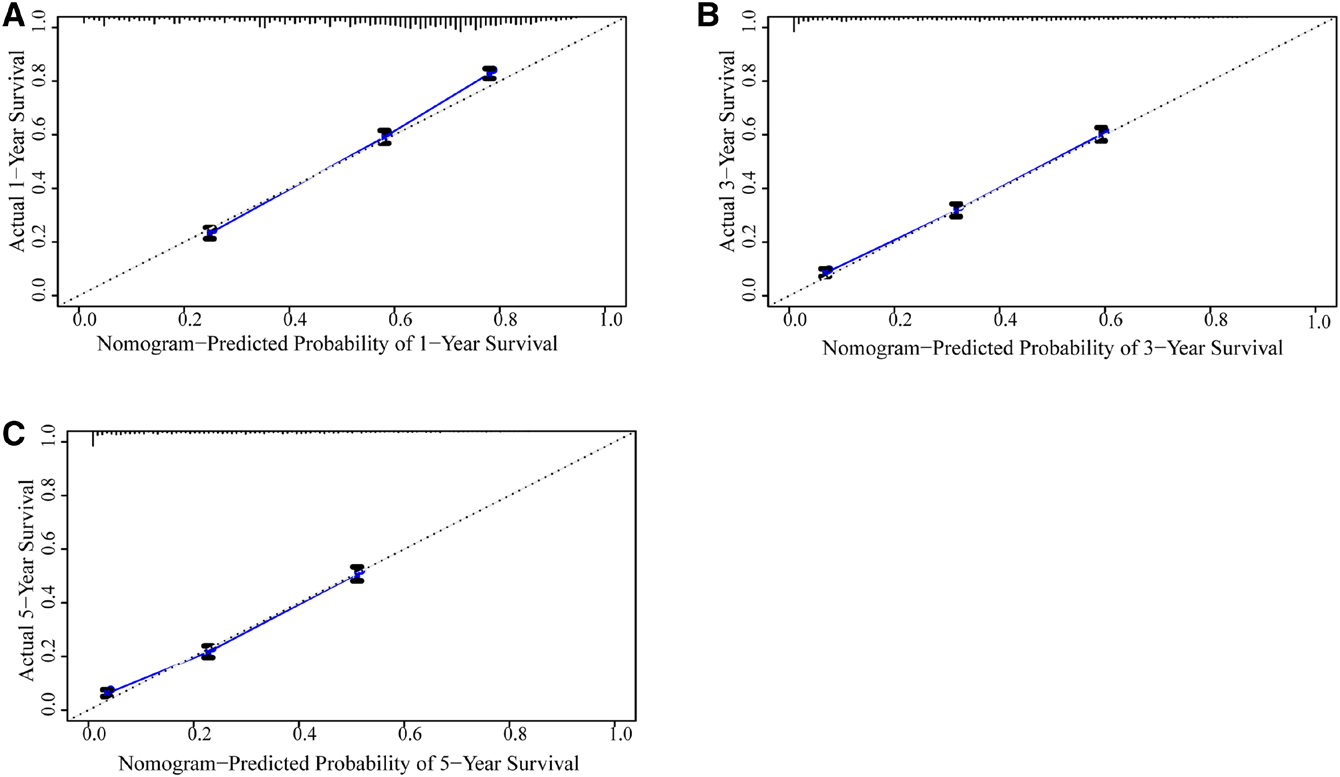
The calibration curve for predicting patient survival. (A) 1-year survival. (B) 3-year survival. (C) 5-year survival.

As shown in Figure 4, the prognostic nomogram integrated all significant independent factors affecting OS. Each variable was scored and the scores were summed to obtain the total score, which provided the survival rate. The C-index of the nomogram for OS was 0.74 (95% CI: 0.73–0.75). The nomogram showed a good prediction accuracy. Next, we performed a calibration analysis (Fig. 5), which showed optimal agreement between the predictions by the nomogram and actual observations.

**Figure 6.**
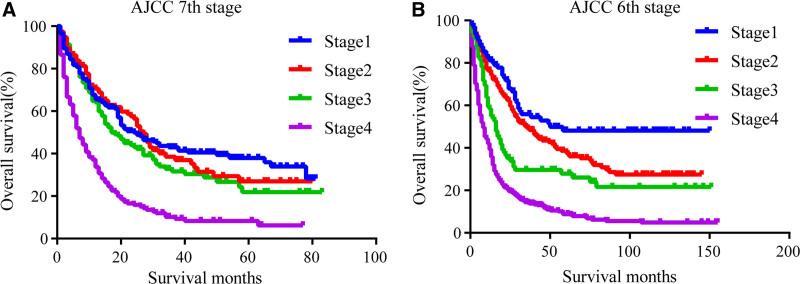
Kaplan–Meier survival curves of the angiosarcoma. (A) AJCC 7th edition. (B) AJCC 6th edition. AJCC = American Joint Committee on Cancer.

We then compared the prognostic accuracy of OS between the conventional staging systems and the nomogram. The AJCC 7th edition was satisfactory with regard to stratifying patients into stage IV and other stages, yet the system was unsatisfactory in stratifying patients into stages I, II, and III (Fig. 6A). The 6th edition of the AJC showed poor prognostic stratification for patients between stages II and III (Fig. 6B). We then compared the C-index values of the nomogram and the AJCC staging system. The C-index of the nomogram was significantly higher than that of the AJCC 6th edition and the AJCC 7th edition (0.74 vs 0.66 vs 0.61, respectively), which showed the accuracy and superiority of the nomogram.

## 4. Discussion

There are few published data on angiosarcoma owing to its rarity; therefore, researchers have not studied its incidence, prevalence, and survival outcomes in detail. To the best of our knowledge, this study had the largest study population to analyze trends in the incidence, prevalence, and survival outcomes of angiosarcoma patients. To date, information on the incidence and prevalence of angiosarcoma has been reported, although some studies have focused on a single site.^[19–22]^ Based on the SEER database, the incidence of angiosarcoma has gradually increased. The age-adjusted annual incidence has increased from 0.13/100,000 in 1975 to 0.33/100,000 in 2016, representing a 2.54-fold increase. To analyze the incidence in detail, we investigated the effects of sex, age, race, site, grade, and stage. According to our study, both males and females showed similar rates of increase, indicating no sex-based differences in the disease. To understand the age distribution of this disease, we divided the patients into 3 groups. We found that the main factor contributing to the incidence rate of the disease was the large increase in the population of people aged 60 years. We found that both White and Black populations showed increased rates, and the age-adjusted rate of the White population was much higher than that of the Black population. Next, we analyzed the site distribution of the disease. The top 6 sites were soft tissue, including the heart, skin excluding basal and squamous, breast, liver, bone and joints, and lung and bronchus. Studies have shown that tumor grade and stage are related to prognosis.^[23,24]^ To further understand the changes in the degree of malignancy of the disease, we analyzed the trends in pathological grade and stage. However, to our surprise, the incidence of G3 and G4 disease was higher than that of G1 and G2, whereas the incidence of local disease was higher than that of regional disease and distant metastasis, suggesting that the grade and stage are not completely synchronized in angiosarcoma. To the best of our knowledge, this is the first analysis of the annual changes in the incidence rate. We evaluated the increasing limited-duration prevalence rates to highlight the burden of angiosarcomas. Prevalence rates are a composite of survival rates and incidence because prevalence rates include not only patients who are undergoing treatment but also patients who have been cured. We found that the increased prevalence rates were mainly attributable to angiosarcoma of the soft tissues, including the heart, skin, and breast.

However, the survival rate of patients with angiosarcoma is poor. Some studies have reported 5-year survival rates of 39.7% and a median OS of 3.4 years in patients with soft tissue and cutaneous angiosarcoma.^[25,26]^ A study revealed that the 3- and 5-year survival rates were 64% and 51%, respectively, in patients with primary breast angiosarcoma.^[27]^ According to a large nationwide cohort study from the Netherlands Cancer Registry, the overall 1-, 2-, and 5-year survival rates are 52.6%, 35.7%, and 21.9%, respectively.^[28]^ In our study, the overall 1-, 3-, and 5-year survival rates were 55.2%, 33.7%, and 26.4%, respectively, which is consistent with the results of previous studies. To visually show the differences in survival of patients with angiosarcoma at different sites, we constructed a histogram. Compared to angiosarcoma at other sites, patients with angiosarcoma of the breast had the best survival, whereas those with angiosarcoma of the liver had the worst survival. Survival is affected by several factors. Wang et al^[29]^ found that tumor differentiation and recurrence or metastasis were independent prognostic factors. A meta-analysis found that prognosis could be affected by age, tumor size, tumor site, and/or surgery. Age <70 years, size <5 cm, face location (vs scalp), and surgery significantly improved the prognosis of patients with scalp and face angiosarcoma.^[30]^ According to another study from the Mayo Clinic, younger age, use of radiation therapy, and multimodal therapy were independent prognostic factors for improved recurrence-free survival and locoregional control, whereas tumor size, grade, margin status, satellite lesions, nodal disease, and surgery showed no correlation with survival.^[31]^ In our study, multivariate analysis showed that age, sex, marital status, grade, historical stage, surgery, site, and tumor size were independent prognostic factors for angiosarcoma. Older age was associated with a worse prognosis, and female patients had significantly better survival. Grades and historical stages were used as the clinical indicators. Smaller tumor size and surgical treatment were associated with better OS in this cohort, which is consistent with the results of previous studies.^[32,33]^ Any inconsistency with other results was probably because different cohorts and clinical factors were included in the analyses. Different primary sites have their own features; therefore, different proportions of patients with angiosarcoma at various sites may lead to different results. Additionally, patients with angiosarcoma may have other diseases that affect their outcomes.

A nomogram is a graphical algorithm used to calculate the probability of an outcome that benefits clinicians by predicting survival more precisely. Patient scores were obtained based on the corresponding indicators, and the total scores were used to assess prognosis. Compared to traditional staging systems, nomograms usually contain more prognostic variables.^[34]^ Nomograms have been developed and have shown high accuracy in predicting the prognosis of somatic cancers.^[35,36]^ Based on the results of multivariate analysis, we included 8 independent prognostic factors to establish a nomogram to predict prognosis: age, sex, marital status, grade, historical stage, surgery, site, and tumor size. The nomogram shows the factors affecting patient prognosis. As shown in the nomogram, age, stage, and site were essential prognostic factors. Patients aged ≥60 years have a worse prognosis than younger patients. Historical stage is an important prognostic predictor. The nomogram showed that patients with liver lesions were more likely to die than those with angiosarcomas at other sites. The C-index of the nomogram was 0.74, indicating that this model could accurately predict the prognosis. Moreover, model calibration was used to predict the agreement between predicted and observed outcomes.^[37]^ Calibration analysis confirmed the association between the actual observed survival rates and nomogram-predicted survival rates. In addition, we compared prognostic accuracy with that of the AJCC staging system. The C-index of our nomogram was significantly better than those of the 6th and 7th editions of the AJCC staging system, indicating the superiority of our nomogram. After calculating the total points on the nomogram, clinicians can make more appropriate treatment choices based on patient expectations.

Our study has several limitations. First, some data were missing. Details regarding the grade were unknown for more than 50% of the patients, which may have influenced the accuracy of the annual age-adjusted incidence of angiosarcoma by grade. In addition, some treatment factors such as the quality of surgery, chemotherapy, and radiation therapy were unavailable in this study and may have confounded the results. These are essential pieces of information to evaluate the effects of treatment on survival. Finally, the nomogram was constructed based on data obtained from White and Black populations, and has not been validated in other populations. Whether this finding is more generalizable remains to be determined.

## 5. Conclusion

We analyzed the largest population to date and presented a comprehensive epidemiological picture of angiosarcomas. The incidence and prevalence of angiosarcoma have increased in recent years. We established a nomogram to predict OS in patients with angiosarcoma, which could greatly benefit clinicians in clinical practice.

## Author contributions

**Conceptualization:** Xi Cao.

**Formal analysis:** Dong Zeng, Zhiyi Wang.

**Investigation:** Yongdong Feng.

**Methodology:** Dong Zeng, Zhiyi Wang.

**Project administration:** Haixia Long, Xi Cao.

**Software:** Dong Zeng.

**Supervision:** Xi Cao.

**Validation:** Dong Zeng.

**Visualization:** Michael J. McKay.

**Writing – original draft:** Dong Zeng.

**Writing – review & editing:** Dong Zeng, Michael J. McKay, Monika K. Masanam, Haixia Long, Xi Cao.

## Supplementary Material



## References

[1] Roberto S Riccardo N Sokol S. Cutaneous angiosarcoma. JAMA Otolaryngol Head Neck Surg. 2024;150:746–8. 38958936 10.1001/jamaoto.2024.1715

[2] Florou V Wilky BA. Current and future directions for angiosarcoma therapy. Curr Treat Options Oncol. 2018;19:14. 29520447 10.1007/s11864-018-0531-3

[3] Goerdt LV Schneider SW Booken N. Cutaneous angiosarcomas: molecular pathogenesis guides novel therapeutic approaches. J Dtsch Dermatol Ges. 2022;20:429–44. 10.1111/ddg.14694 35218306

[4] Ishida Y Otsuka A Kabashima K. Cutaneous angiosarcoma: update on biology and latest treatment. Curr Opin Oncol. 2018;30:107–12. 29194075 10.1097/CCO.0000000000000427 PMC5815647

[5] Gervais MK Burtenshaw SM Maxwell J. c. J Surg Oncol. 2017;116:1056–61. 29205355 10.1002/jso.24780

[6] Koo J Knight-Perry J Galambos C Browne LP Cost CR. Pediatric metastatic cardiac angiosarcoma successfully treated with multimodal therapy: case report and review of literature. J Pediatr Hematol Oncol. 2021;43:e203–6. 31725539 10.1097/MPH.0000000000001674

[7] Edwards D Voronina A Attwood K Grand'Maison A. Association between occupational exposures and sarcoma incidence and mortality: systematic review and meta-analysis. Syst Rev. 2021;10:231. 34389054 10.1186/s13643-021-01769-4 PMC8364027

[8] Juin Hsien BL Shelat VG. Spleen angiosarcoma: a world review. Expert Rev Gastroenterol Hepatol. 2021;15:1115–41. 34160346 10.1080/17474124.2021.1945920

[9] An R Men XJ Ni XH Wang W-T Wang C-L. Angiosarcoma of the breast: A review. Heliyon. 2024;10:e24413. 38318005 10.1016/j.heliyon.2024.e24413 PMC10839862

[10] Alves VAF Rimola J. Malignant vascular tumors of the liver in adults. Semin Liver Dis. 2019;39:1–12. 30536289 10.1055/s-0038-1676120

[11] Sturm EC Marasco IS Katz SC. Multidisciplinary management of angiosarcoma e a review. J Surg Res. 2021;257:213–20. 32858322 10.1016/j.jss.2020.07.026

[12] Tsuneki M Kinjo T Mori T. Survivin: a novel marker and potential therapeutic target for human angiosarcoma. Cancer Sci. 2017;108:2295–305. 28845553 10.1111/cas.13379 PMC5665764

[13] Wang M Wu S Tong A Cui X Ma X. The prognostic value of pretreatment inflammatory biomarkers in primary angiosarcoma. Cancer Manag Res. 2019;11:7981–9. 31686912 10.2147/CMAR.S219237 PMC6709794

[14] Kim H Choi N Baek CH Son Y-I Jeong H-S Chung MK. Comparison of prognostic implications between the 7th and 8th edition of AJCC TNM staging system for head and neck soft-tissue sarcoma in adult patients. Eur Arch Otorhinolaryngol. 2019;276:3195–202. 31399768 10.1007/s00405-019-05584-5

[15] Doll KM Rademaker A Sosa JA. Practical guide to surgical data sets: surveillance, epidemiology, and end results (SEER) database. JAMA Surg. 2018;153:588–9. 29617544 10.1001/jamasurg.2018.0501

[16] Rosen PP Kimmel M Ernsberger D. Mammary angiosarcoma. The prognostic significance of tumor differentiation. Cancer. 1988;62:2145–51. 3179927 10.1002/1097-0142(19881115)62:10<2145::aid-cncr2820621014>3.0.co;2-o

[17] Ojamaa K Innos K Baburin A Everaus H Veerus P. Trends in cervical cancer incidence and survival in Estonia from 1995 to 2014. BMC Cancer. 2018;18:1075. 30404606 10.1186/s12885-018-5006-1 PMC6222998

[18] Lu Z Yan W Liang J. Nomogram based on systemic immune-inflammation index to predict survival of tongue cancer patients who underwent cervical dissection. Front Oncol. 2020;10:341. 32219070 10.3389/fonc.2020.00341 PMC7078378

[19] Sato F Yamamoto T. Breast angiosarcoma after primary breast cancer surgery: a systematic review. J Plast Reconstr Aesthet Surg. 2022;75:2882–9. 35907689 10.1016/j.bjps.2022.06.046

[20] Ramakrishnan N Mokhtari R Charville GW Bui N Ganjoo K. Cutaneous angiosarcoma of the head and neck-a retrospective analysis of 47 patients. Cancers (Basel). 2022;14:3841. 35954504 10.3390/cancers14153841 PMC9367417

[21] Wang X Lu Z Luo Y. Characteristics and outcomes of primary pleural angiosarcoma: a retrospective study of 43 published cases. Medicine (Baltim). 2022;101:e28785. 10.1097/MD.0000000000028785 PMC8830823 35147108

[22] Zhang C Huang C Zhang X Zhao L Pan D. Clinical characteristics associated with primary cardiac angiosarcoma outcomes: a surveillance, epidemiology and end result analysis. Eur J Med Res. 2019;24:29. 31426842 10.1186/s40001-019-0389-2 PMC6699122

[23] Yuan WH Li AF Hsu HC Hu Y-S Lee R-C. Initial clinical radiological findings and staging to predict prognosis of primary hepatic angiosarcoma: a retrospective analysis. PLoS One. 2019;14:e0225043. 31710641 10.1371/journal.pone.0225043 PMC6844487

[24] Ito T Uchi H Nakahara T. Cutaneous angiosarcoma of the head and face: a single-center analysis of treatment outcomes in 43 patients in Japan. J Cancer Res Clin Oncol. 2016;142:1387–94. 27015673 10.1007/s00432-016-2151-2 PMC11819220

[25] Bi S Zhong A Yin X Li J Cen Y Chen J. Management of cutaneous angiosarcoma: an update review. Curr Treat Options Oncol. 2022;23:137–54. 35182299 10.1007/s11864-021-00933-1

[26] Sinnamon AJ Neuwirth MG McMillan MT. A prognostic model for resectable soft tissue and cutaneous angiosarcoma. J Surg Oncol. 2016;114:557–63. 27378102 10.1002/jso.24352

[27] Abdou Y Elkhanany A Attwood K Ji W Takabe K Opyrchal M. Primary and secondary breast angiosarcoma: single center report and a meta-analysis. Breast Cancer Res Treat. 2019;178:523–33. 31522347 10.1007/s10549-019-05432-4 PMC6817750

[28] Weidema ME Flucke UE van der Graaf WTA; Dutch Nationwide Network and Registry of Histo- and Cytopathology (PALGA)-Group. Prognostic factors in a large nationwide cohort of histologically confirmed primary and secondary angiosarcomas. Cancers (Basel). 2019;11:1780. 31726650 10.3390/cancers11111780 PMC6896046

[29] Wang L Lao IW Yu L Wang J. Clinicopathological features and prognostic factors in angiosarcoma: a retrospective analysis of 200 patients from a single Chinese medical institute. Oncol Lett. 2017;14:5370–8. 29113171 10.3892/ol.2017.6892 PMC5656021

[30] Shin JY Roh SG Lee NH Yang K-M. Predisposing factors for poor prognosis of angiosarcoma of the scalp and face: systematic review and meta-analysis. Head Neck. 2017;39:380–6. 27507124 10.1002/hed.24554

[31] Patel SH Hayden RE Hinni ML. Angiosarcoma of the scalp and face: the Mayo Clinic experience. JAMA Otolaryngol Head Neck Surg. 2015;141:335–40. 25634014 10.1001/jamaoto.2014.3584

[32] Lindet C Neuville A Penel N. Localised angiosarcomas: the identification of prognostic factors and analysis of treatment impact. A retrospective analysis from the French Sarcoma Group (GSF/GETO). Eur J Cancer. 2013;49:369–76. 22967726 10.1016/j.ejca.2012.08.016

[33] Lahat G Dhuka AR Hallevi H. Angiosarcoma: clinical and molecular insights. Ann Surg. 2010;251:1098–106. 20485141 10.1097/SLA.0b013e3181dbb75a

[34] Liu W Wang Z Wu Y Li L. Establishment and assessment of a nomogram for predicting prognosis in bone-metastatic prostate cancer. Medicine (Baltim). 2023;102:e35693. 10.1097/MD.0000000000035693 PMC10627693 37933039

[35] Boakye D Jansen L Schneider M Chang-Claude J Hoffmeister M Brenner H. Personalizing the prediction of colorectal cancer prognosis by incorporating comorbidities and functional status into prognostic nomograms. Cancers (Basel). 2019;11:1435. 31561507 10.3390/cancers11101435 PMC6826360

[36] Fornaro L Vivaldi C Parnofiello A. Validated clinico-pathologic nomogram in the prediction of HER2 status in gastro-oesophageal cancer. Br J Cancer. 2019;120:522–6. 30745584 10.1038/s41416-019-0399-4 PMC6461920

[37] Changhee L Alexander L Ahmed A. Application of a novel machine learning framework for predicting non-metastatic prostate cancer-specific mortality in men using the surveillance, epidemiology, and end results (SEER) database. Lancet Digit Health. 2021;3:158–65. 10.1016/S2589-7500(20)30314-9 33549512

