# Supplementary figures and images for: Trends in the incidence, survival, and prognostic nomogram of angiosarcoma in the United States
Source: Medicine (Baltimore). 2025 Jan 3;104(1):e41152. doi: 10.1097/MD.0000000000041152 (PMC11709153; doi:10.1097/MD.0000000000041152)

Supplementary Figure S1

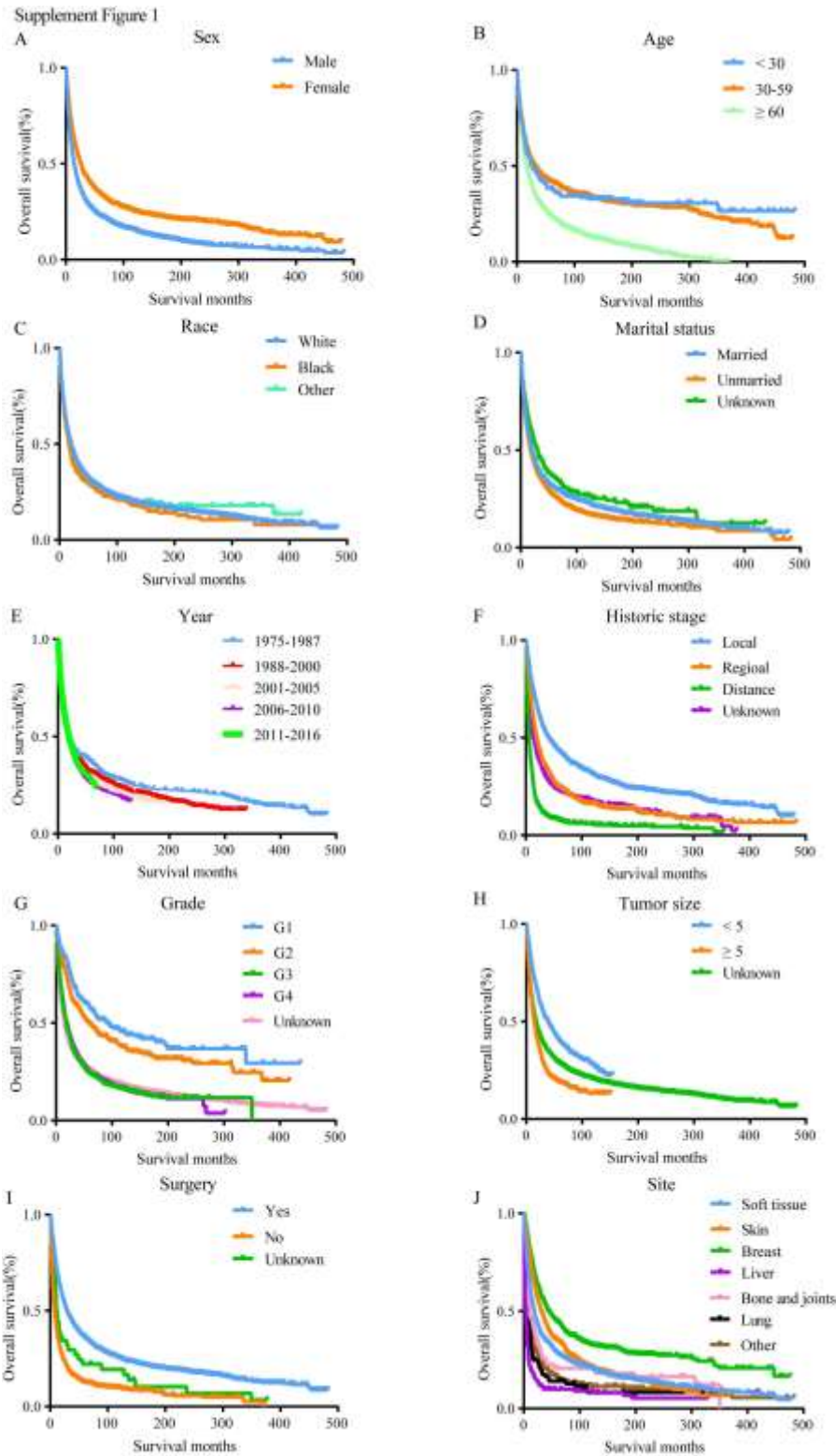

Supplement: Supplementary file 1 [file medi-104-e41152-s001.pdf]
